# Ultrasound Measured Depth of Pelvic Free Fluid Correlates Well with Blood Loss Volume in Patients with Ectopic Pregnancy

**DOI:** 10.1155/2020/8874581

**Published:** 2020-12-10

**Authors:** Shuai Tang, Qing Zhou, Yuelun Zhang, Lin Chen, Xuerong Yu, Yanming Zhang, Zhenhong Qi, Yu Xia, Yuguang Huang

**Affiliations:** ^1^Department of Anesthesiology, Peking Union Medical College Hospital, CAMS & PUMC, Beijing, China; ^2^Department of Ultrasound, Peking Union Medical College Hospital, CAMS & PUMC, Beijing, China

## Abstract

**Purpose:**

The ultrasonic finding of pelvic free fluid which suggests the possibility of internal haemorrhage helps the determination of the severity of patients.

**Methods:**

We conducted a retrospective study investigating ultrasonic measurements and haemorrhage volumes in patients having an ectopic pregnancy in a single centre from January 2013 to November 2016. The logistic regression model was used to establish the prediction model for haemorrhage volumes. The diagnostic accuracy was evaluated by area under ROC curve (AUC) analysis. We employed 800 ml as the cut-off point of the haemorrhage and further set it to 1000 ml and 1200 ml in the sensitivity analysis.

**Results:**

The mean pelvic free fluid depths measured by TVS and TAS were 4.45 ± 2.15 cm and 4.45 ± 2.56 cm in the haemorrhage ≥800 ml group, while they were 2.48 ± 1.51 cm and 2.55 ± 1.19 cm in <800 ml group. AUCs and the corresponding cut-off points were 0.741 (95% CI 0.677 to 0.804) and 0.118 when predicted by the standardised depths of TVS and TAS, 0.784 (95% CI 0.696–0.872) and 2.95 cm by the raw depths of TVS, and 0.748 (95% CI 0.665–0.831) and 3.35 cm by the raw depths of TAS.

**Conclusions:**

The depth of pelvic free fluid measured by TVS and TAS can be used to predict blood loss volume in patients having an ectopic pregnancy. TVS may perform better than TAS.

## 1. Introduction

Ectopic pregnancy (EP) is the implantation of the fertilised ovum outside the endometrial cavity of uterine. It occurred in approximately 1.0–2.0% of pregnancies and increased recently due to the advent of assisted reproductive technology [1, 2]. EP usually results in miscarriage. It can cause some severe complications such as loss of future fertility, haemorrhagic shock, and death when ruptured.

EP used to account for 3–4% of pregnancy-related deaths, but the associated mortality has decreased owing to the early diagnosis and treatment before rupture [1, 3, 4]. Ultrasonography, which provides real-time pelvic imaging, has revolutionised the early diagnosis of EP. For patients with EP, the ultrasound examination may find free fluid in peritoneal and pelvic cavities [5]. Compared with the existing clinical parameters (such as blood pressure, hematocrit, and haemoglobin), emergency ultrasonography at the point-of-care ultrasound (POC US) may guide a faster determination of the severity of patients' conditions and lead to expedited care and is thus a critical auxiliary examination. Previous research assessing the relationship between depths of pelvic free fluid measured by ultrasonography and haemorrhage volumes was insufficient. We undertook this research to study whether the depth of pelvic free fluid measured by ultrasound scan, either transabdominally or transvaginally, can predict the haemorrhage amount; thus deriving a hemoperitoneum score in ectopic pregnancy.

## 2. Materials and Methods

We carried out a retrospective study performed at a single centre. We reviewed the medical records of female patients with EP and underwent laparoscopy/laparotomy surgery at Peking Union Medical College Hospital from January 2013 to November 2016. Among them, patients who underwent preoperative ultrasonography to measure the depths of pelvic free fluid and had the blood loss volume recorded were included in the analysis. All data were collected anonymously from medical records and were nonidentifiable. The Ethics Committee of the Chinese Academy of Medical Sciences and the Peking Union Medical College Hospital has approved this study and agreed to waive the written informed consents on 5 November 2018.

The primary outcome was blood loss volume, and the predictor is the superoinferior (S/I) depth of pelvic free fluid measured by transvaginal ultrasound scan (TVS) or anteroposterior (AP) depth measured by transabdominal ultrasound scan (TAS). Historically, more than 50% of the patients whose haemorrhage ≥800 ml received an autologous blood transfusion during surgery. Therefore, we chose 800 ml as the cut-off point and divided the haemorrhage into two groups: ≥800 ml and <800 ml. Potential confounders included age, BMI, hypertension, surgery procedures, and previous abdominal or pelvic surgery. We further set the diagnostic cut-off point to 1000 ml and 1200 ml in sensitivity analyses.

We employed standardised depths of TAS or TVS instead of raw data in binary logistic regression analysis. Standardized depths were calculated using the raw measured depth minus the mean of the depth and divided by the standard deviation. We used the standardised depths, rather than the raw measured depth, because depths measured by TVS and TAS cannot be compared directly. By standardization, the exponential coefficient from the regression model can be interpreted as the increased probability of ≥800 ml with each standard deviation in the independent variable. BMI and age were also included in the models, because they may affect the whole blood volume of patients. We set up three models, calculated OR, and *p* values of variables in each model and compared the ORs of standardised depths (depth for short in the model) between three models. If In(OR) of depth fluctuated within 10%, indicating OR was stable, the most simplified model was chosen to demonstrate the relationship between depth and blood loss volume.

  Model 1 : haemorrhage∼*β*0  +  *β*1*∗*depth
  Model 2 : haemorrhage∼*β*0  +  *β*1*∗*depth + *β*2*∗*BMI
  Model  3 : haemorrhage∼*β*0  +  *β*1*∗*depth + *β*2*∗*BMI  +  *β*3*∗*age.

Statistical analysis was as follows. Variables were tested by independent sample *t*-test or chi-square test. ROC curves were used to evaluate the accuracy of the depths when predicting an 800 ml haemorrhage volume. The area under the ROC curve (AUC), optimal diagnostic cut-off point, sensitivity, specificity, positive predictive value (PPV), and negative predictive value (NPV) were calculated. SPSS version 22 was used in all statistical analyses. The two-sided probability of type I error was 0.05 in the analysis.

## 3. Results

A total of 581 patients were diagnosed with EP and underwent emergent or selective surgery. The blood loss volume was obtained from the operation note of 393 patients. Among them, 281 cases were available for the documented ultrasound measurements in our centre. Generally, the types of ultrasound scan were determined by the physicians according to the availability and doctors' preference. Patients with the ultrasonography done in other hospitals were not included in the analysis to reduce measurement bias.

The overall mean age was 30.93 ± 5.45 years, and the overall mean BMI was 21.80 ± 3.63 kg/m^2^, the mean blood loss volume was 727.15 ± 850.80 ml, and 261/281 (93.2%) of the cases in this study were classified as ASA physical status I-II. 136/281 (48.4%) underwent TVS, and the mean depth of pelvic free fluid was 2.88 ± 1.83 cm, while 150/281 (53.4%) underwent TAS, and the mean depth was 3.35 ± 2.10 cm, respectively.

Patients enrolled were divided into haemorrhage <800 ml group and haemorrhage ≥800 ml group. Means and proportions of the two groups are compared in Table 1. Statistically significant differences were noted between groups regarding the mean haemorrhage volume, mean depths of pelvic free fluid evaluated by both TVS and TAS, ASA physical status, abdominal pain, mean preoperative and postoperative haemoglobin, fluid infusion, and blood products transfusion. There were no statistically significant differences in the *p* value of the potential confounders including age (*p*=0.917), BMI (*p*=0.606 to 1.000 in different BMI groups), surgery procedures (*p*=0.321), and previous abdominal or pelvic surgery (*p*=0.710). The mean depths were roughly the same within groups (2.48 ± 1.51 cm by TVS and 2.55 ± 1.19 cm by TAS in haemorrhage <800 ml group, 4.45 ± 2.15 cm by TVS and 4.45 ± 2.56 cm by TAS in haemorrhage ≥800 ml group).

The OR (95% CI) and *p* values of the covariates in three models were calculated (Table 2). Only the depth was statistically significant (*p* value <0.001). OR values of depth among these models were between 2.6 (95% CI 1.7 to 4.1) and 2.9 (95% CI 1.8 to 4.6), suggesting that the association was stable. As stated in the methodological section, model 1 was selected to illustrate the relation of pelvic free fluid depth and haemorrhage volume in the following analyses.

**Figure 1:**
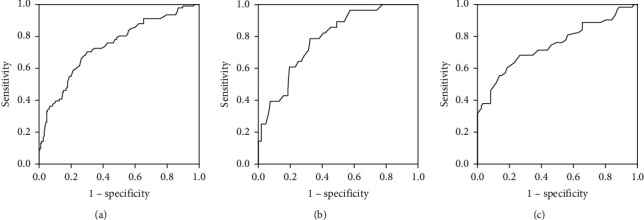
ROC curves of haemorrhage volumes predicted by pelvic free fluid depths. The haemorrhage volumes were predicted by standardised pelvic free fluid depths evaluated by TVS and TAS in panel a, by the raw depth of TVS in panel b and raw depths of TAS in panel c separately. TAS: transabdominal ultrasonography; TVS: transvaginal Ultrasonography; Se: sensitivity; Sp: specificity. (a) Haemorrhage volume predicted by standardised depth measured by TVS and TAS, (b) haemorrhage volume predicted by raw depth measured by TVS, and (c) haemorrhage volume predicted by raw depth measured by TAS.

Figure 1 shows the ROC curve of haemorrhage volume predicted by standardised pelvic free fluid depth evaluated by TVS and TAS (panel a), and by the raw depth of TVS (panel b) or TAS (panel c) separately. AUC of panel a was 0.741 (95% CI 0.677 to 0.804), and the corresponding diagnostic cut-off point was 0.118 (the corresponding raw depth measured by TVS was 2.83 cm and by TAS was 3.46 cm). AUC of panel b was 0.784 (95% CI 0.696–0.872), and the corresponding cut-off point was 2.95 cm, while AUC of panel c was 0.748 (95% CI 0.665–0.831), and the corresponding cut-off point was 3.35 cm, respectively. Although AUC of panel b was the largest, AUC between three panels fluctuated within 10%.

In the subsequent sensitivity analyses, we increased the threshold of haemorrhage to 1000 ml and 1200 ml and calculated the AUC of different types of ultrasonography. The AUCs for TVS were 0.814 (haemorrhage ≥1000 ml) and 0.903 (haemorrhage ≥1200 ml), significantly higher than that of TAS (0.780 and 0.742), indicating that TVS may be more accurate than TAS in the case of larger haemorrhage volume.

## 4. Discussion

Results from our study demonstrated that the depth of pelvic free fluid measured by either TAS or TVS could be used to estimate the haemorrhage volume for patients with EP, thus proposing a hemoperitoneum score for cell salvage use in ectopic pregnancy. The diagnostic cut-off point of pelvic free fluid depth for haemorrhage ≥800 ml was 2.95 cm measured by TVS and 3.35 cm by TAS.

Ultrasonography was previously proved to be sensitive and accurate for the diagnosis of EP [6–8]. Prior research also indicated that the presence of a moderate-to-large amount of pelvic free fluid significantly increased the level of suspicion for tubal rupture of EP [5, 9, 10], while a small amount of free fluid are associated with healthy pregnancies [11]. However, the ultrasonic criteria to predict the amount of intraperitoneal blood varied in previous research, which was partially ascribed to the heterogeneity of the US measurement.

TVS and TAS have already been widely applied for gynaecological examination and diagnosis. It is commonly believed that TVS has some advantages over TAS. We initially attempted to compare the predictive accuracies of TVS and TAS within the same patients. However, there were only six cases with both TVS and TAS measurements that were infeasible to provide convincing results. Our research hence compared the AUCs of TVS or TAS of different patients and found them approximately equal when predicting haemorrhage volume at a cut-off point of 800 ml. TVS performed better at higher blood loss threshold (1000 ml and 1200 ml) in the subsequent sensitivity analyses. The result was consistent with our clinical experience. The probe of TVS is more proximal to pelvic organs and able to bypass obstacles such as gas-filled bowels or adhesions in the pelvic cavity so that TVS provides better image resolution and accuracy. In urgent situations, TVS is more time saving because patients do not need waiting for bladder filling for visualisation of TAS.

Ultrasonography is a noninvasive method to estimate the amount of intraperitoneal blood due to ruptured ectopic pregnancy. With the wide application of POS US, the ultrasonic scan provides faster diagnosis and condition assessments of suspected EP patients rather than the existing laboratory parameters such as hematocrit and haemoglobin. The risk determination may contribute to initiating appropriate treatments including emergency surgical management and perioperative patient care. Patients with a possibly massive bleeding or hemodynamic instability require colloid infusion and blood transfusion to alleviate haemorrhagic shock. Previous research showed that, for EP patients with massive haemoperitoneum, intraoperative autologous blood transfusion was a safe and feasible procedure. Cell salvage can reduce homologous blood transfusion without lengthening hospitalization [12–15]. Autologous blood transfusion can reduce the risk of allergies, immunosuppression, acute lung injury, and other transfusion-related complications. It also saves blood resources and decreases the economic burden on society [16]. The 2009 Association of Anaesthetists of Great Britain and Ireland (AAGBI) guidelines recommended patients with anticipated blood loss of >1000 ml, low haemoglobin, or increased risk of bleeding to consider using intraoperative cell salvage. Taking into account the risk of continuous bleeding in EP patients, we chose 800 ml as a reasonable threshold to adopt cell salvage or homologous blood transfusion. It was consistent with our results: the proportions of patients with blood transfusion (homologous or autologous) in blood loss ≥800 ml and <800 ml groups were 53/91 (58.2%) and 2/190 (1.1%), respectively, and cell salvage accounted for 51/55 (92.7%) among these cases. In addition to blood transfusion therapy, as the medical practices is a coshared responsibility with the surgery, anaesthesiology, radiology, and emergency medicine, the risk determination also contributes to the relevant decision-making of other medical professionals to initiate proper treatments and care.

The heterogeneity of the US measurement limited the reproducibility and reliability of our results. The single ultrasonic scan of patients cannot predict the blood loss volumes in a dynamic bleeding process. The depths obtained in our results tended to overestimate the amount of blood loss due to the time intervals from ultrasonography to operation. Another limitation lies in the choice of the one-dimensional depths as the predictors to assess the blood volumes distributed in the three-dimensional body cavities. Observer bias is also concerned in single measurements, so peer reviews of ultrasonic results are recommended in the study design. Further research can collect US measurements at different times to establish a dynamic predicting model of blood loss volumes. As POC US facilitates the preoperative US measurements in the operating room, it can be widened in the subsequent research to provide more timely and accurate data of intraperitoneal blood.

In conclusion, the depth of pelvic free fluid measured by TVS and TAS can be used to estimate blood loss volume in patients clinically suspected with EP. The optimal diagnostic cut-off point was 2.95 cm for TVS and 3.35 cm for TAS when estimating whether blood loss volume was more than 800 ml and indicate the employment of cell salvage instead of homologous transfusion. TVS may perform better than TAS, when haemorrhage volume is higher.

## Figures and Tables

**Table 1:** Clinical characteristics of the ectopic pregnancy patients.

Characteristic	Haemorrhage <800 ml (*N* = 190)	Haemorrhage ≥800 ml (*N* = 91)
	Number (%) or mean ± SD		*p* value	Difference or OR (95% CI)
Age-yr.	30.91 ± 5.19	30.98 ± 5.99	0.917	−0.7 (−1.4 to 1.3)
BMI^*a*^, no. (%)				
≤18.4	26 (13.7)	12 (13.2)	—	—
18.5–23.9	120 (63.2)	51 (56.0)	0.831	0.9 (0.4 to 2.0)
24.0–27.9	29 (15.3)	17 (18.7)	0.606	1.3 (0.5 to 3.2)
≥28.0	10 (5.3)	4 (4.4)	1.000	0.9 (0.2 to 3.3)
ASA^*b*^ class, no. (%)				
I	123 (64.7)	27 (29.7)	—	—
II	64 (33.7)	47 (51.6)	<0.001	3.3 (1.9 to 5.9)
III	2 (1.1)	16 (17.6)	<0.001	36.4 (7.9 to 167.9)
IV	0 (0.0)	1 (1.1)	0.185	13.5 (0.5 to 339.6)
Mean volume of haemorrhage, ml	263.84 ± 187.93	1694.51 ± 882.34	<0.001	−1430.7 (−1562.3 to −1299.0)
Previous abdominal or pelvic surgery, no. (%)	101 (40.2)	49 (44.5)	0.710	1.1 (0.7 to 1.8)
Abdominal pain, no. (%)	197 (78.5)	106 (96.4)	0.007	6.1 (1.4 to 26.84)
Ultrasonography				
Types				
TVS^*c*^ only, no. (%)	102 (53.7)	28 (30.8)	—	—
TAS^*d*^ only, no. (%)	82 (43.2)	63 (69.2)	<0.001	2.8 (1.6 to 4.8)
Both, no. (%)	6 (3.2)	0 (0.0)	0.345	0.3 (0.0 to 5.1)
Depth				
Superoinferior depth of hemoperitoneum by TVS, cm	2.48 ± 1.51	4.45 ± 2.15	<0.001	−2.0 (−2.7 to −1.3)
Anteroposterior depth of hemoperitoneum by TAS, cm	2.55 ± 1.19	4.45 ± 2.56	<0.001	−1.9 (−2.5 to −1.3)
Surgery procedure, no. (%)				
Laparoscopy	188 (98.9)	88 (96.7)	—	—
Laparotomy	0 (0.0)	1 (1.1)	0.321	6.4 (0.3 to 158.4)
Fluids infusion				
Crystoloid solution infusion, ml	1086.84 ± 368.88	1259.34 ± 510.71	0.001	−172.5 (−277.9 to −67.1)
Colloid solution infusion, ml	178.95 ± 261.41	648.35 ± 383.65	<0.001	−469.4 (−546.3 to −392.6)
Blood products				
RBC^*e*^ transfusion, no. (%)	0 (0.0)	13 (14.3)	<0.001	65.5 (3.8 to 1115.8)
Plasma transfusion, no. (%)	0 (0.0)	7 (7.7)	<0.001	32.0 (1.8 to 566.4)
Cell salvage, no. (%)	2 (1.1)	49 (53.8)	<0.001	109.7 (25.6 to 468.9)
Preoperative haemoglobin (pre-Hgb^*f*^), g/L	118.56 ± 13.09	101.48 ± 14.84	<0.001	17.1 (13.6 to 20.5)
Postoperative haemoglobin (post-Hgb), g/L	104.93 ± 12.50	90.48 ± 15.54	<0.001	14.4 (10.9 to 18.0)
Post-Hgb–Pre-Hgb, g/L	−13.33 ± 10.03	−11.42 ± 16.17	0.248	−1.9 (−5.2 to 1.3)

*a*: BMI, body mass index; *b*: ASA, American Society of Anesthesiologists; *c*: TVS, transvaginal ultrasound; *d*: TAS, transabdominal ultrasound; *e*: RBC, red blood cell; *f*: Hgb, haemoglobin.

**Table 2:** Prediction model of haemorrhage volume by depth, BMI, and age.

Models	OR (95% CI)	*p* value
Model 1		
Hemorrhage∼*β*0 + *β*1*∗*depth^*a*^	2.6 (1.7 to 4.1)	<0.001
Depth		
Model 2		
Hemorrhage∼*β*0 + *β*1*∗*depth + *β*2*∗*BMI^*b*^	2.8 (1.8 to 4.5)	<0.001
Depth		
BMI	1.1 (1.0 to 1.2)	0.275
Model 3		
Haemorrhage∼*β*0 + *β*1*∗*depth + *β*2*∗*BMI + *β*3*∗*age	<0.001	
Depth	2.9 (1.8 to 4.6)	
BMI	1.1 (1.0 to 1.2)	0.243
Age	1.0 (0.9 to 1.1)	0.344

*a*: depth: the standardised depths measured by transvaginal ultrasound scan and transabdominal ultrasound scan; *b*: BMI, body mass index.

## Data Availability

The datasets generated and analysed during the current study are not publicly available due to patients' privacy but are available from the corresponding author on reasonable request.

## Abbreviations

AAGBI: Association of Anaesthetists of Great Britain and Ireland
ASA: American Society of Anesthesiologists
AUC: Area under ROC curve
BMI: Body mass index
CI: Confidence interval
EP: Ectopic pregnancy
NPV: Negative predictive value
OR: Odds ratio
POC US: Point-of-care ultrasound
PPV: Positive predictive value
ROC: Receiver operating characteristic
TAS: Transabdominal ultrasonography
TVS: Transvaginal ultrasonography.

## References

[1] Chang J., Elam-Evans L. D., Berg C. J. (2003). Pregnancy-related mortality surveillance—United States, 1991–1999. *MMWR Surveillance Summaries*.

[2] Saraiya M., Berg C. J., Shulman H., Green C. A., Atrash H. K. (1999). Estimates of the annual number of clinically recognized pregnancies in the United States, 1981–1991. *American Journal of Epidemiology*.

[3] Berg C. J., Callaghan W. M., Syverson C., Henderson Z. (2010). Pregnancy-related mortality in the United States, 1998 to 2005. *Obstetrics & Gynecology*.

[4] Creanga A. A., Shapiro-Mendoza C. K., Bish C. L., Zane S., Berg C. J., Callaghan W. M. (2011). Trends in ectopic pregnancy mortality in the United States. *Obstetrics & Gynecology*.

[5] Frates M. C., Doubilet P. M., Peters H. E., Benson C. B. (2014). Adnexal sonographic findings in ectopic pregnancy and their correlation with tubal rupture and human chorionic gonadotropin levels. *Journal of Ultrasound in Medicine*.

[6] Barnhart K., Mennuti M., Benjamin I., Jacobson S., Goodman D., Coutifaris C. (1994). Prompt diagnosis of ectopic pregnancy in an emergency department setting. *Obstetrics & Gynecology*.

[7] Shalev E., Yarom I., Bustan M., Weiner E., Ben-Shlomo I. (1998). Transvaginal sonography as the ultimate diagnostic tool for the management of ectopic pregnancy: experience with 840 cases. *Fertility and Sterility*.

[8] Condous G., Lu C., Van Huffel S. V., Timmerman D., Bourne T. (2004). Human chorionic gonadotrophin and progesterone levels in pregnancies of unknown location. *International Journal of Gynecology & Obstetrics*.

[9] Mol B., Hajenius P., Engelsbel S. (1999). Can noninvasive diagnostic tools predict tubal rupture or active bleeding in patients with tubal pregnancy?. *Fertility and Sterility*.

[10] Frates M. C., Brown D. L., Doubilet P. M., Hornstein M. D. (1994). Tubal rupture in patients with ectopic pregnancy: diagnosis with transvaginal US. *Radiology*.

[11] Gurel S., Sarikaya B., Gurel K., Akata D. (2007). Role of sonography in the diagnosis of ectopic pregnancy. *Journal of Clinical Ultrasound*.

[12] Banks A., Norris A. (2005). Massive haemorrhage in pregnancy. *Continuing Education in Anaesthesia Critical Care & Pain*.

[13] Uchil D. (2015). Cell salvage in laparoscopic surgery for ectopic pregnancy with massive hemoperitoneum. *Journal of Minimally Invasive Gynecology*.

[14] Yamada T., Okamoto Y., Kasamatsu H., Mori H. (2003). Intraoperative autologous blood transfusion for hemoperitoneum resulting from ectopic pregnancy or ovarian bleeding during laparoscopic surgery. *Journal of the Society of Laparoendoscopic Surgeons*.

[15] Selo-Ojeme D. O., Onwudiegwu U., Durosinmi M. A., Owolabi A. T. (1997). Emergency autologous blood transfusion in the management of ruptured ectopic pregnancy. *Journal of Obstetrics and Gynaecology*.

[16] Davies L., Brown T., Haynes S., Payne K., Elliott R., McCollum C. (2006). Cost-effectiveness of cell salvage and alternative methods of minimising perioperative allogeneic blood transfusion: a systematic review and economic model. *Health Technol Assess*.

